# Antimicrobial Photosensitizing Material Based on Conjugated Zn(II) Porphyrins

**DOI:** 10.3390/antibiotics11010091

**Published:** 2022-01-12

**Authors:** Sofía C. Santamarina, Daniel A. Heredia, Andrés M. Durantini, Edgardo N. Durantini

**Affiliations:** IDAS-CONICET, Departamento de Química, Facultad de Ciencias Exactas, Físico-Químicas y Naturales, Universidad Nacional de Río Cuarto, Ruta Nacional 36 Km 601, Río Cuarto, Córdoba X5804BYA, Argentina; ssantamarina@exa.unrc.edu.ar (S.C.S.); dheredia@exa.unrc.edu.ar (D.A.H.); adurantini@exa.unrc.edu.ar (A.M.D.)

**Keywords:** polymer, porphyrin, photosensitizer, antimicrobial, photodynamic inactivation

## Abstract

The widespread use of antibiotics has led to a considerable increase in the resistance of microorganisms to these agents. Consequently, it is imminent to establish new strategies to combat pathogens. An alternative involves the development of photoactive polymers that represent an interesting strategy to kill microbes and maintain aseptic surfaces. In this sense, a conjugated polymer (PZnTEP) based on Zn(II) 5,10,15,20-tetrakis-[4-(ethynyl)phenyl]porphyrin (ZnTEP) was obtained by the homocoupling reaction of terminal alkyne groups. PZnTEP exhibits a microporous structure with high surface areas allowing better interaction with bacteria. The UV-visible absorption spectra show the Soret and Q bands of PZnTEP red-shifted by about 18 nm compared to those of the monomer. Also, the conjugate presents the two red emission bands, characteristic of porphyrins. This polymer was able to produce singlet molecular oxygen and superoxide radical anion in the presence of NADH. Photocytotoxic activity sensitized by PZnTEP was investigated in bacterial suspensions. No viable *Staphylococcus aureus* cells were detected using 0.5 µM PZnTEP and 15 min irradiation. Under these conditions, complete photoinactivation of *Escherichia coli* was observed in the presence of 100 mM KI. Likewise, no survival was detected for *E. coli* incubated with 1.0 µM PZnTEP after 30 min irradiation. Furthermore, polylactic acid surfaces coated with PZnTEP were able to kill efficiently these bacteria. This surface can be reused for at least three photoinactivation cycles. Therefore, this conjugated photodynamic polymer is an interesting antimicrobial photoactive material for designing and developing self-sterilizing surfaces.

## 1. Introduction

The emergence of microbes resistant to antibiotics denotes a continuing threat to public health throughout the world [1,2]. Several factors are responsible for mutagenic changes in pathogenic microbes, such as the trend towards the use of broad-spectrum antibiotics, the applications of antimicrobials in hospitals as a prophylactic measure, the inappropriate prescription of drugs, self-medication, and the non-compliance of treatment patients [3]. In addition, the resistance of microorganisms can increase due to the use of antibiotics for multiple purposes in livestock farming [4]. Antibiotic contamination is increasing rapidly in the natural environment. In particular, inadequate wastewater treatment strategies in various settings facilitate the evolution and spread of antibiotic-resistant microbes in the effluents that supply water reservoirs [5].

Even though a wide variety of microorganisms can be produced diseases, some bacteria are mainly responsible for infections acquired in health centers [6]. Thus, the Gram-positive bacterium *Staphylococcus aureus* represents one of the pathogens with the highest epidemiological resistance found in hospitals [7]. This microorganism is considered a serious threat to human health care. Additionally, the Gram-negative strains of *Escherichia coli* commenced to acquire resistance to predictable antibiotics [8]. The vast majority of *E. coli* isolated from human extra-intestinal clinical infections are now multidrug-resistant. In this way, diarrheogenic *E. coli* is one of the leading causes of foodborne illness associated with intestinal diseases [9]. Consequently, it is of crucial relevance to develop alternative therapies to eliminate resistant pathogens and thus prevent a future health crisis. New actions are imperative, at both the community and hospital levels, to improve the diagnosis and treatment of bacterial infections. Therefore, it is essential to establish alternative strategies to promote the elimination of these resistant bacterial strains from the environment [10,11].

In this sense, antimicrobial photodynamic inactivation (PDI) has been projected as a promising therapy to eradicate pathogens [12]. This approach is founded on the addition of a photosensitizer (PS) that binds selectively to microbial cells. Light irradiation of appropriate wavelengths in aerobiosis leads to the formation of reactive oxygen species (ROS). Subsequently, these ROS can produce damage to microbial cells, including oxidation of membrane lipids, nucleic acids, amino acids, and cross-linking of proteins [13]. The main reactions depend on the location of the PS in the cells. Therefore, when a PS binds to the envelope of microorganisms, oxidative damage to proteins and fatty acids at the localization site is expected due to the high reactivity, short lifetime, and limited diffusion of the ROS in this microenvironment. These molecular alterations produce a loss of biological functionality, leading to microbial death. The photodynamic effect induced by the PS can mainly occur through two competitive mechanisms [14]. In the type I process, the PS excited triplet state reacts with biological molecules by electron or proton transfer, forming free radicals. These highly reactive intermediates can interact with molecular oxygen to produce ROS, such as hydroxyl radical (HO^•^), superoxide anion radical (O_2_^•−^), and hydrogen peroxide (H_2_O_2_). In type II pathway, the PS excited triplet state produces energy transfer to molecular oxygen, generating singlet molecular oxygen, O_2_(^1^Δ_g_) [15].

Several potential photosensitizing compounds have been used to produce the inactivation of microbes [16,17,18]. The most commonly used PS are those derived from porphyrins [16,19]. However, unsubstituted porphyrins are generally insoluble in polar organic solvents and exhibit high aggregation in aqueous media due to the intrinsic planarity of the tetrapyrrolic macrocycle. These characteristics limit the complete application of these PSs in PDI. Therefore, the substitution in the periphery of the macrocycle by groups that allows for the improvement of the interaction with biological media is a promising alternative for medical applications [12,19]. Another possibility is to obtain conjugated polymers formed by repeating porphyrin units [20,21]. These porous photoactive materials can be used to photoinactivate microorganisms suspended in liquid media, attached to a support, and coating self-sterilizing surfaces. In addition, organic porous materials can facilitate the interaction of constituent PSs with microorganisms, improving the photoinactivating capacity [22]. Surfaces containing immobilized porphyrins were projected for the inactivation of microorganisms, taking into account economic and ecological topics [23]. In this sense, coating of surfaces with PSs is of great interest for maintaining aseptic surfaces in healthcare environments.

**Scheme 1:**
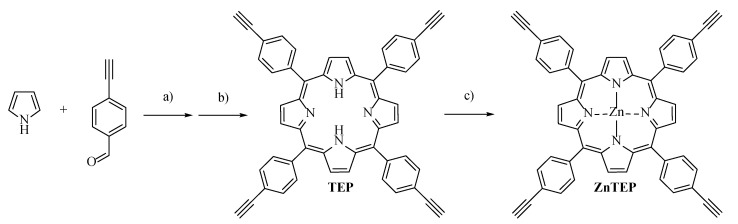
Synthesis of ZnTEP. Reagents and conditons: (**a**) BF_3_.OEt_2_, DCM, r.t., 3 h; (**b**) DDQ, DCM, r. t., 2 h, 34%; (**c**) Zn(CH_3_COO)_2_, DCM/MeOH, 18 h, 98%.

**Scheme 2:**
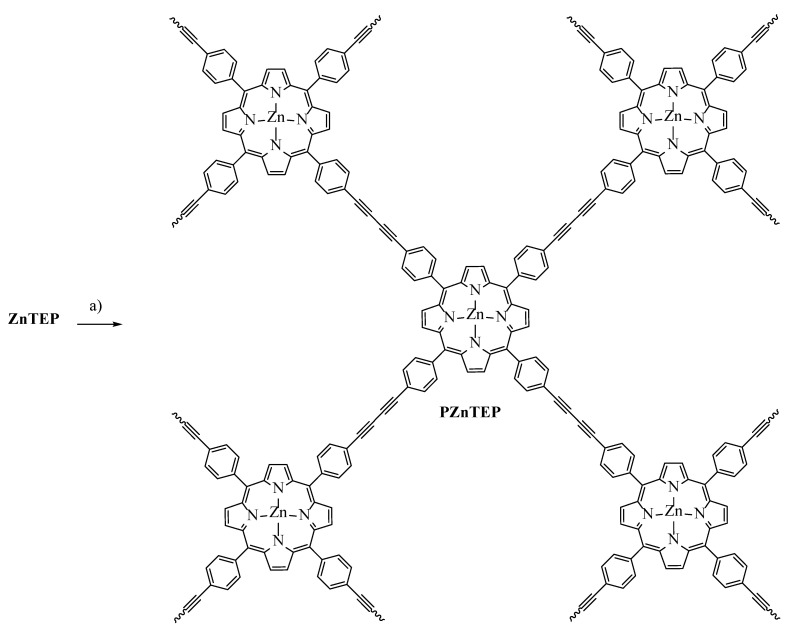
Synthesis of PZnTEP. Reagents and conditions: (**a**) CuI, PdCl_2_(PPh_3_)_2_, TEA, THF, r.t., 48 h, 99%.

In this work, ZnTEP porphyrin was used as the modular unit to build the conjugated polymer PZnTEP by a homocoupling reaction of terminal alkynes (Scheme 1 and Scheme 2). The polymeric material was analyzed by scanning electron microscopy (SEM). Spectroscopic characteristics of this polymer were compared with those of its constitutional monomer in solution. Also, photodynamic properties were investigated in the presence of different molecular probes to detect the formation of ROS. The application of PDI was studied in cells suspensions of *S. aureus* and *E. coli*. The effect of PDI in combination with KI was also evaluated to eliminate the Gram-negative pathogenic microorganism. Furthermore, the polymer PZnTEP was embedded in polylactic acid (PLA) surfaces. This photodynamic material was assessed as self-sterilizing surfaces to inactivate bacteria.

## 2. Materials and Methods

Instrumentation and supplies are described in Supplementary Materials.

### 2.1. Synthetic Procedures

Synthesis of 5,10,15,20-tetrakis-[4-(ethynyl)phenyl]porphyrin (TEP). 4-(Ethinyl)benzaldehyde (260 mg, 2.0 mmol) and pyrrole (140 μL, 2.0 mmol) were dissolved in dichloromethane (DCM, 350 mL). The resulting solution was purged with argon for 15 min and then BF_3_.OEt_2_ (70 μL) was added. The reaction mixture was stirred for 3 h at room temperature. After that, 2,3-dichloro-5,6-dicyano-1,4-benzoquinone (DDQ, 340 mg, 1.5 mmol) was added and the solution was stirred for 2 h at room temperature. The organic solvent was eliminated under reduced pressure and the crude solid was chromatographed (silica gel, *n*-hexane/DCM (7:3)) to afford the free-base porphyrin TEP (121 mg, 34%). ^1^H-NMR (CDCl_3_, TMS) δ (ppm)−2.80 (brs, 2H, pyrrole NH), 3.37 (s, 4H, C≡CH,), 7.94 (d, 8H, *J* = 8.00 Hz, 3,5-ArH,), 8.19 (d, 8H, *J* = 8.00 Hz, 2,6-ArH), 8.88 (s, brs, pyrrole-H, 8H). ESI-MS (m/z) 711.2556 [M + H]^+^ (711.2549 calculated for [M + H]^+^, M = C_52_H_30_N_4_).

Synthesis of Zn(II) 5,10,15,20-tetrakis-[4-(ethynyl)phenyl]porphyrin (ZnTEP). A saturated solution of Zn(II) acetate (5 mL) in methanol was added to a solution of TEP (30 mg, 0.042 mmol) in DCM (10 mL). The resulting suspension was stirred overnight at room temperature. Then, the reaction mixture was washed with water (3 × 15 mL). The organic solvent was removed under reduced pressure to give the metalated porphyrin ZnTEP (32 mg, 98%). ^1^H-NMR (CDCl_3_, TMS) δ (ppm) 3.39 (s, 4H, C≡CH,), 7.92 (d, 8H, *J* = 8.00 Hz, 3.5-ArH,), 8.18 (d, 8H, *J* = 8.00 Hz, 2,6-ArH), 8.84 (s, brs, pyrrole-H, 8H). ESI-MS (m/z) 773.1693 (773.1684 calculated for [M + H]^+^, M = C_52_H_28_N_4_Zn).

Synthesis of polymeric ZnTEP (PZnTEP). To a solution of ZnTEP (20 mg, 26 µmol) in dry tetrahydrofuran (THF, 1.1 mL) was added PdCl_2_(PPh_3_)_2_ (2 mg, 0.3 μmol) and CuI (1 mg, 5 μmol) under argon atmosphere. The resulting mixture was sonicated until dissolution was achieved. After that, triethylamine (TEA, 21 μL) was added and the solution was kept in dark without stirring at room temperature for 48 h. The reaction crude was washed with THF to give the desired organogel PZnTEP in a quantitative yield. The xerogel of PZnTEP was prepared by drying under high vacuum a portion of the organogel (Figure S1A).

### 2.2. Spectroscopic Determinations

UV-visible absorption and fluorescence spectra were acquired in *N*,*N*-dimethylformamide (DMF). Determinations were performed with a quartz cell of 1 cm path length at room temperature. Fluorescence emission spectra were determined by exciting the solutions at 565 nm. At this wavelength, the absorbances of the samples were 0.05 and the emission spectra were integrated in the range between 600 and 800 nm. The fluorescence quantum yield (Φ_F_) of the porphyrins was calculated from the area below the corrected emission spectrum using Zn(II) 5,10,15,20-tetra(4-methoxyphenyl)porphyrin (ZnTMP) as a reference (Φ_F_ = 0.049) [24].

### 2.3. Photooxidation of 9,10-Dimethylanthracene (DMA)

Solutions of DMA (35 µM) and PS (A = 0.1 at 565 nm) were prepared in 2 mL of DMF. Samples were exposed to light at 455–800 nm (44 mW/cm^2^, Figure S2A) The photooxidation rate of DMA was analyzed by determining the decrease in absorbance at 378 nm (Figure S3). Pseudo-first order kinetic plots of ln(A_0_/A) vs. time were used to obtain the values of the observed rate constant of DMA (*k*_obs_^DMA^). Quantum yields of O_2_(^1^Δ_g_) production (Φ_Δ_) were calculated by comparing the *k*_obs_^DMA^ for the corresponding PS with that for ZnTMP, which was used as a reference (Φ_Δ_ = 0.73) [24].

### 2.4. Photoreduction of Nitro Blue Tetrazolium (NBT)

The NBT method was used to detect the formation of O_2_^•−^ [25]. This approach was carried out using 0.2 mM NBT, 0.5 mM NADH and PS (A = 0.1 at 565 nm) in 2 mL of DMF/water (5% *v*/*v*). Control experiments were performed in absence of PS. Samples were irradiated under aerobic conditions with light at 455–800 nm (44 mW/cm^2^, Figure S2A). The progress of the reaction was monitored by following the increase of the absorbance at λ = 560 nm (Figure S4) [26].

### 2.5. Bacterial Strains and Growth Conditions

Stock cultures of the strains methicillin-resistant *S. aureus* (ATCC 43300) and *E. coli* (ATCC 25922) were kept in glycerol 10% (*v*/*v*) and tryptic soy (TS) broth 90% (*v*/*v*) at −80 °C. All bacterial strains were grown in TS agar at 37 °C for 20 h. Then, an aliquot (400 µL) of the bacterial culture was aseptically transferred to 4 mL of fresh TS broth and incubated at 37 °C until reaching the exponential phase of growth (A = 0.4 at 660 nm for *S. aureus* and A = 0.6 at 660 nm for *E. coli*). Cells were centrifuged (3000 rpm for 15 min) and re-suspended in an equal amount of PBS, corresponding to ~10^8^ colony forming units (CFU)/mL. After that, cells were diluted 1/100 in PBS to obtain ~10^6^ CFU/ mL. Viable bacteria were quantified by the spread plate technique using serial dilutions 10-fold in PBS. Each sample was streaked on TS agar plates in triplicate. The formation of colonies was counted after incubation of 24 h at 37 °C in the dark.

### 2.6. Photosensitized Inactivation of Bacterial Suspensions

Cell suspensions of bacteria (2 mL, ~10^8^ UFC/mL) in PBS were incubated with 0.5 and 1.0 µM PZnTEP for *S. aureus* and 1.0 µM PZnTEP for *E. coli* in Pyrex culture tubes (13 × 100 mm) for 30 min in the dark at 37 °C. PZnTEP was added from a stock solution 0.5 mM in DMF (Figure S1B). Then, 200 mL of each cell suspension was transferred to 96-well microtiter plates. Cells were exposed for different time intervals (15 and 30 min, which represent 81 and 162 J/cm^2^, respectively) to white light (90 mW/cm^2^, Figure S2B,C). For PDI tests of *E. coli* in presence of KI, before adding PZnTEP (1.0 µM) the cell suspensions (2mL, 10^8^ CFU/mL) were previously treated with 100 mM KI for 30 min at 37 °C in the dark [27]. KI was added from an aqueous 1.0 M solution. After that, *E. coli* cells were irradiated as mentioned above for 30 min (162 J/cm^2^). Viable cells were determined as described above.

### 2.7. Photosensitized Inactivation of Bacteria with PZnTEP Adsorbed on a PLA Surface

The polymer PZnTEP was deposited on the PLA surface by spin coating as previously described [28]. Briefly, a thin layer of PLA (0.05 mm) was 3D-printed on a coverslip (24 × 24 mm). Then, it was placed on a heating plate at 215 °C for 3 min to smooth the surface. A solution (200 μL, 0.5 mM) of PZnTEP in DCM/THF (1:1) was added dropwise on the surface previously mounted on a spin coater operating at 3000 rpm under ambient conditions. The dropping distance was 10 cm. After that, the sample remained spinning for 1 min. This methodology allowed to disperse homogeneously a solution of the PZnTEP on the PLA surface that remained permanently adsorbed after evaporation of the solvent. After that, the sample chamber was ensembled by gluing with silicon a 3D-printed cylinder (ø = 18 mm) on top of the coated coverslip (Figure S1C,D). The final volume of the chamber was 1 mL. Bacterial suspensions were prepared following the methodology described above. An inoculum of 300 µL of *S. aureus* and *E. coli* (~10^6^ CFU/mL) were placed inside the chamber. After that, the cells were exposed to white light for 30 min (162 J/cm^2^). Cell viability after each assay was quantified as previously indicated. After each PDI experiment, the chamber was cleaned with PBS and reutilized in successive experiments.

### 2.8. Statistical Analysis

Bacterial controls were attained using irradiated cultures without the porphyrin polymer and in the presence of PZnTEP in the dark. Each value represents the mean of three separate experiments and the error bar denotes the standard deviation. Statistically significant meanings were established by one-way ANOVA. Values were statistically significant considering a confidence level of 95% (*p* < 0.05).

## 3. Results and Discussion

### 3.1. Synthesis of ZnTEP and PZnTEP

The synthetic pathway to prepare the Zn(II) porphyrin monomer containing terminal alkynyl groups (ZnTEP) is outlined in Scheme 1. First, 4-(ethynyl)benzaldehyde and pyrrole were subjected to an acid-catalyzed condensation in DCM at room temperature for 3 h, affording the hydrogenated macrocycle. Oxidation of the latter with DDQ gave rise to porphyrin TEP in 34% yield as a purple solid after a simple purification by flash column chromatography. This free-base porphyrin was then metaled by treating TEP with Zn(II) acetate in DCM/methanol at room temperature to give the desired metal complex ZnTEP in 98% yield. These two synthetic steps provide in good yields the photoactive monomer with four ethynylphenyl moieties around the macrocycle.

Having an efficient synthetic route toward the ZnTEP building block, the next task was to assess appropriate conditions for the preparation of the conjugated material by the polymerization of diynes. For this purpose, the reported conditions that involve a homocoupling reaction of terminal alkynes were adequate to generate the desired polymeric conjugate [29]. ZnTEP in anhydrous THF was treated with TEA, CuI, and PdCl_2_(PPh_3_)_2_ (Scheme 2). After 48 h, the reaction afforded the polymeric material PZnTEP as a black organogel in a quantitative yield. The product was subjected to a simple purification by washing with THF to obtain the conjugated polymer organogel. Thus, PZnTEP, obtained through the carbon-carbon coupling reactions of the terminal alkynyls, is a porous material that was then dried under high vacuum to prepare the xerogel [29].

### 3.2. SEM Images of the Polymer

**Figure 1:**
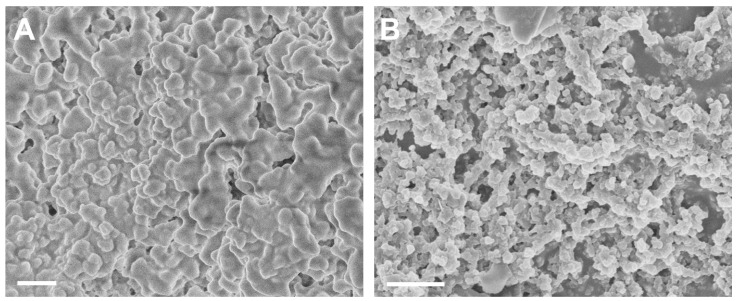
SEM images of PZnTEP polymeric material (**A**) PZnTEP organogel deposited as a film on glass and (**B**) a portion of PZnTEP xerogel, scale bar 1 µm.

The conjugated polymer PZnTEP was analyzed by SEM images, as shown in Figure 1. To this end, an aliquot of the organogel polymer in THF was spread on a glass surface to form a film. Them, the solvent was allowed to evaporate at room temperature. Figure 1A displays a representative SEM image of this material. This image presents a structure with overlapping scales covering the surface. A portion of the polymer was dried under high vacuum to obtain xerogel polymeric. An illustrative SEM image of this material is exposed in Figure 1B. The PZnTEP polymer as xerogel exhibits porous structures due to the removal of THF trapped in the interstices of the conjugated material as organogel. Thus, the xerogel polymer retains its original shape with a more contracted material shape and a particle size ranging from 200 nm.

It was previously found that porphyrin-based organic polymer also includes micropores in its structure [22]. In addition, a polymer based on covalently connected porphyrin with 4-thiophenephenyl groups showed many flat nanosheets [30]. Polymer of metalloporphyrin exhibited the rough surface, revealing a porous structure [31]. In this way, aromatic frameworks based on 5,10,15,20-tetraphenylporphyrin exhibited irregular shapes with high surface areas [32]. In our case, the synthesized porous material offers a larger contact surface, which can be beneficial for PDI of bacteria.

### 3.3. Absorption and Fluorescence Spectroscopic Characterization

**Figure 2:**
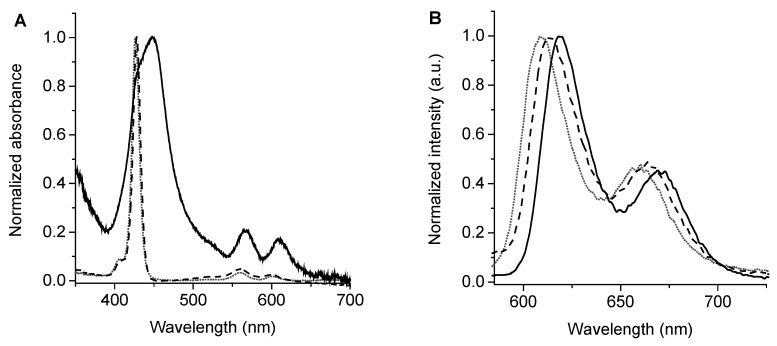
(**A**) UV-visible absorption and (**B**) fluorescence emission spectra of PZnTEP (solid line), ZnTEP (dashed line), and ZnTMP (dotted line) in DMF (λ_exc_ = 565 nm).

Figure 2A shows the UV-visible absorption spectra of PZnTEP and ZnTEP in DMF. In addition, the optical properties of these compounds were compared with ZnTMP. Table 1 summarizes the spectroscopic characteristics of these porphyrin derivatives. The spectra of monomers ZnTEP and ZnTMP exhibit a Soret band at ∼ 427 nm with high molar absorption coefficient (10^5^ Lmol^−1^cm^−1^) and two less intense Q bands between 500 and 600 nm. These bands are typical of the *meso*-substituted porphyrins that complex with Zn(II) [24,33]. Furthermore, the absorption of PZnTEP indicates that the spectroscopic properties of the porphyrin unit (ZnTEP) were kept in the polymeric material, despite being a widely conjugated system. In addition, the UV-visible absorption results confirm the polymerization of ZnTEP as the constitutional component of the PZnTEP conjugate. Moreover, the Soret and Q bands of PZnTEP show a bathochromic shift relative to ZnTEP. In particular, the Sored band displays a red-shifted maximum of 18 nm in this medium. Also, a broadening of both bands was observed in the polymer, indicating a slight interaction between the Zn(II) porphyrin units in the conjugated structure [23].

Figure 2B shows the fluorescence emission spectra of ZnTEP and ZnTMP in DMF. Two bands centered at ∼ 610 and ∼ 670 nm were found, which are representative of *meso*-substituted Zn(II) porphyrins [24,33]. The emission bands correspond to the Q_x_(0-0) and Q_x_(0-1) transitions that are distinctive of porphyrins with a D_2h_ symmetry. Thus, the vibronic structure of these compounds remains almost unchanged upon excitation [34]. On the other hand, the emission spectrum of PZnTEP exhibits an emission band positioned at 620 nm and a less intense band at 680 nm. These bands are bathochromically shifted compared to ZnTEP. This behavior is consistent with the shift observed in the Q bands of the polymer. Furthermore, PZnTEP showed adequate emission properties, indicating that the fluorescence characteristics of the porphyrin unit were mainly retained in the polymer. Therefore, ZnTEP was conjugated in the polymeric material without considerable aggregation. Small changes in the absorbance and fluorescence spectra of PZnTEP indicate that the π-π stacking between the porphyrin units was hindered and only occurs a weak interaction. Additionally, the Stokes shift for the polymer was calculated from the absorption and fluorescence wavelength maxima of the Q_x_(0-0) band. A Stokes shift of ∼11 nm for PZnTEP indicates that small structural changes occur between the ground state and the excited singlet state of porphyrin, due to the rigid structure of the tetrapyrrolic macrocycle. Values of Φ_F_ were determined using ZnTMP as a reference (Table 1) [24]. The Φ_F_ value for PZnTEP was 6 and 4-fold smaller than ZnTEP and the reference, respectively. The results for ZnTEP and PZnTEP agree with those previously reported for similar structures forming complexes with Zn(II) [33].

### 3.4. Production of O_2_(^1^Δ_g_)

**Figure 3:**
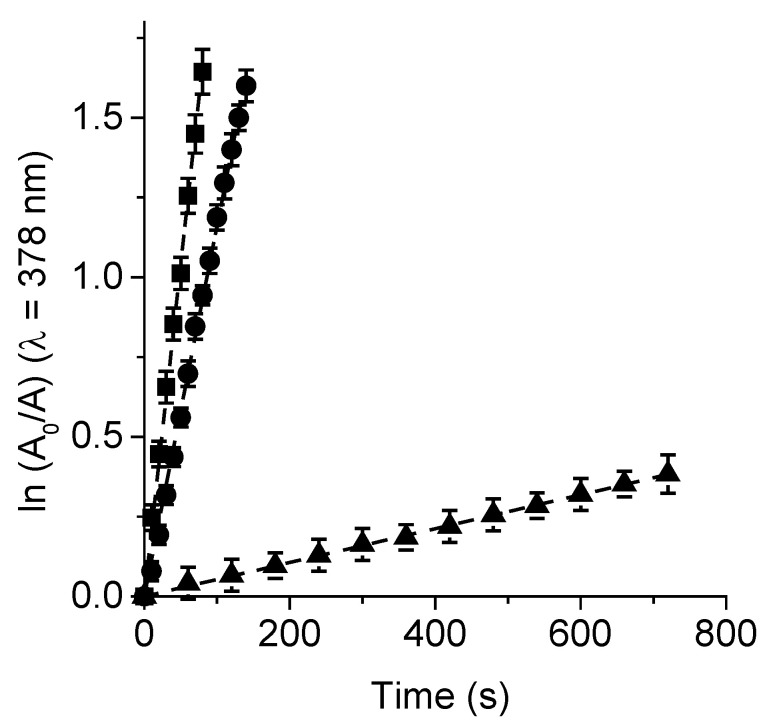
First-order plots for the photooxidation of DMA sensitized by ZnTMP (⯀), ZnTEP (●) and PznTEP (▲) in DMF, λ_irr_ = 455–800 nm.

Photodecomposition of DMA sensitized by ZnTEP and PZnTEP was determined in DMF. The solutions of this anthracene derivative under aerobic conditions were exposed to light between 455 and 800 nm. The photooxidation of DMA was investigated following the changes in the UV-visible absorption spectra (Figure S3). The progress of the reactions was detected by the decrease in the intensity of the DMA band at 378 nm due to the formation of the corresponding 9,10-endoperoxide (Figure S3) [35]. In all cases, reaction rates exhibit a pseudo-first order kinetic at a wavelength of 378 nm with respect to the DMA concentration (Figure 3). Furthermore, the reaction rates obtained for ZnTEP and PZnTEP were compared with those using ZnTMP as a reference [24]. In Table 1 is specified the values of *k*_obs_^DMA^ sensitized by these compounds. As can be observed, the decomposition rate of DMA induced by the PZnTEP polymer is approximately 30-fold lower than that of its monomer.

Considering that DMA quenches O_2_(^1^Δ_g_) by chemical reaction, the values of *k*_obs_^DMA^ were used to determine the capacity of these PSs to form O_2_(^1^Δ_g_) [36]. Therefore, the values of Φ_Δ_ were calculated by comparing the kinetic data of each PS with that of the reference (ZnTMP). The results are summarized in Table 1. The Φ_Δ_ value obtained for ZnTEP was slightly lower than expected for a Zn(II) porphyrin derivative [24,33]. ZnTEP is a low polarity porphyrin and this tetrapyrrolic macrocycle cannot be completely dissolved as a monomer in DMF, reducing the production of O_2_(^1^Δ_g_). Although the value of Φ_Δ_ for the PZnTEP is relatively low compared to the monomer in solution, photooxidation induced by this conjugate can be considered appropriate, since O_2_(^1^Δ_g_) formation occurs in the polymeric structure. Similar outcomes were previously obtained for conjugated polymers of porphyrins embedded in a polymer matrix [23,36]. However, this O_2_(^1^Δ_g_) production may be appropriate to produce a photoinactivating activity of microorganisms if combined with the formation of radicals (type I pathway).

### 3.5. Formation of O_2_^•−^

**Figure 4:**
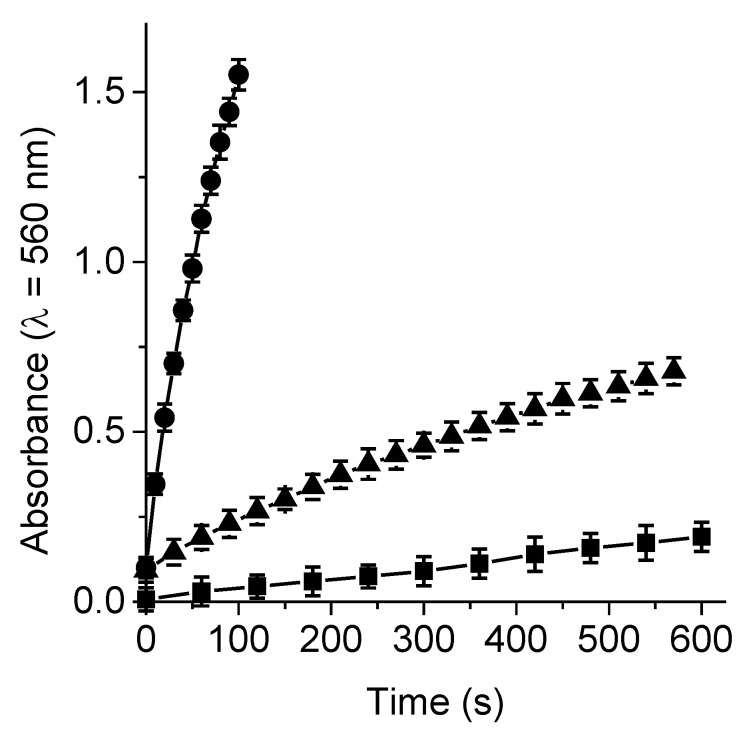
Time course of O_2_^•−^ formation sensed by the NBT approach following the increase in the absorption at 560 nm for NBT + NADH + ZnTEP (●), NBT + NADH + PZnTEP (▲) and NBT + NADH (⯀) in DMF/water (5%), [NBT] = 0.2 mM and [NADH] = 0.5 mM, λ_irr_ = 455–800 nm.

The capacity of ZnTEP and PZnTEP to generate O_2_^•−^ by type I pathway was investigated in DMF/water (5%). For this application, solutions of PS containing NBT and the reducing agent NADH were irradiated with light between 455 and 800 nm under aerobic conditions. The reaction of NBT with O_2_^•−^ produced diformazan that can be monitored following the absorption band of this product, which is centered around 560 nm (Figure S4). The generation of O_2_^•−^ detected by the NBT is shown in Figure 4. These results indicate that both monomeric porphyrin and its conjugated polymer can form O_2_^•−^ efficiently in presence of an electron donor agent, such as NADH. ZnTEP exhibited a marked production of this ROS after short irradiation times. The absorption of diformazan sensitized by PZnTEP was about 4 times higher than the control without photoactive material. In previous investigations, the formation of diformazan was also found for the reaction of NBT sensitized by several porphyrin derivatives [25,37,38]. Therefore, even though ZnTEP and PZnTEP produce O_2_(^1^Δ_g_) in solution, they are also capable of sensitizing the formation of O_2_^•−^. The electron transfer type of reaction that leads to O_2_^•−^ formation preferentially occurs in polar solvents, mainly with the incidence of a reducing agent, such as NADH. In contrast, O_2_(^1^Δ_g_) generation takes place in non-polar microenvironments. These photoprocesses that yield O_2_(^1^Δ_g_) and O_2_^•−^ can be considered as the two main photochemical reaction pathways known as type II and type I mechanisms, respectively. Both mechanisms can act simultaneously and the preponderance of one over the other depends on the PS structure, the polarity of the medium in which it is located and the presence of different substrates in the medium.

### 3.6. Photosensitized Inactivation of Bacteria

**Figure 5:**
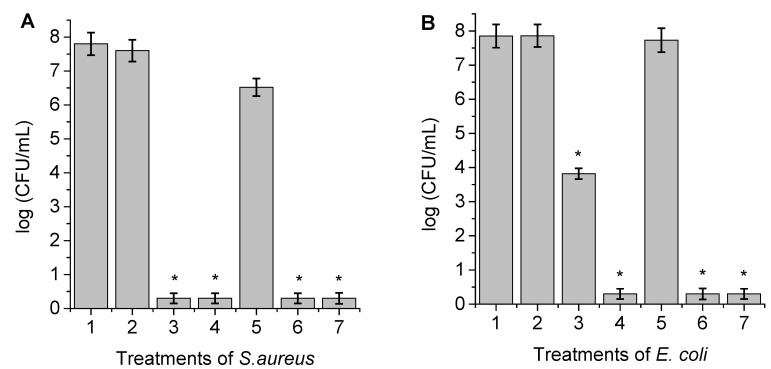
Survival of bacteria (~10^8^ CFU/mL) incubated with PZnTEP for 30 min at 37 °C in the dark and irradiated with white light for different times. (**A**) *S. aureus*: (1) dark control, (2) 0.5 µM PZnTEP in dark, (3) 0.5 µM PZnTEP and 15 min irradiation, (4) 0.5 µM PZnTEP and 30 min irradiation, (5) 1.0 µM PZnTEP in dark, (6) 1.0 µM PZnTEP and 15 min irradiation, (7) 1.0 µM PZnTEP and 30 min irradiation; (**B**) *E. coli*: (1) dark control, (2) 1.0 µM PZnTEP in dark, (3) 1.0 µM PZnTEP and 15 min irradiation, (4) 1.0 µM PZnTEP and 30 min irradiation, (5) 1.0 µM PZnTEP + 100 mM KI in dark, (6) 1.0 µM PZnTEP + 100 mM KI and 15 min irradiation, (7) 1.0 µM PZnTEP + 100 mM KI and 30 min irradiation (* *p* < 0.05 compared with control).

In order to benchmark the quality of PZnTEP as a microbial annihilator, the photodynamic polymeric material was tested to inactivate bacteria. Here, the viability of the cells after exposure to different irradiation periods was assessed in planktonic media. Two strain models were selected the Gram-positive methicillin-resistant *S. aureus* and the Gram-negative *E. coli*. First, bacteria suspended in PBS (10^8^ CFU/mL) were treated with polymer PZnTEP for 30 min at 37 °C in the dark. Subsequently, the cells were exposed to white light for different times (15 and 30 min that corresponds to 81 and 162 J/cm^2^, respectively). *S. aureus* cells were incubated with 0.5 and 1.0 µM PS, while *E. coli* was treated only with 1.0 µM PS. At these concentrations, the dark control only showed slight toxicity (∼1 log) when *S. aureus* was incubated with 1 µM PZnTEP (Figure 5A, treatment 5). Moreover, the viability of the bacteria was not affected by cell irradiation without PS (Figure S5). Therefore, we can ensure that for the following set of experiments photoinactivation of bacteria was caused by the photodynamic effect produced by the conjugated polymer.

As can be appreciated in Figure 5, the polymer PZnTEP has photoinactivating activity achieving a 7.5 log reduction in the CFU for *S. aureus*, after a short period of irradiation (15 min) and low concentration (0.5 µM PZnTEP). This photokilling represents a reduction of bacterial cell survival greater than 99.9999% (Figure 5, treatments 3 and 4). Similar results were observed after 15 and 30 min irradiation using 1.0 µM PZnTEP (Figure 5, treatments 6 and 7). Although this polymer is not soluble in aqueous media, this may favor the interaction of the photodynamic material with the cells of *S. aureus*, increasing the photoinactivating capacity.

On the other hand, *E. coli*, a more resistant and difficult bacteria to eliminate, due to its more complex cell wall, showed a moderate drop of CFU after 15 min irradiation (Figure 5B, treatment 3), producing a 4 log (99.99%) reduction in cell survival. By doubling the treatment time, complete elimination of *E. coli* was observed for cultures treated with a light dose of 162 J/cm^2^ (Figure 5B, treatment 4). The lower inactivation for *E. coli*, relative to S. *aureus,* at the same concentration of PZnTEP, can be explained by considering the structural differences in the cell membrane [17]. Gram-negative bacteria have an outer membrane that functions as an effective permeability barrier between the cell and the surrounding environment, restricting the binding and penetration of many molecules. In general, in vitro studies with microorganisms indicate that Gram-positive strains are susceptible to the effect produced by a wide variety of PSs including those that are neutral or anionic compounds, while Gram-negatives are resistant to several PDI treatments. Therefore, this type of bacteria is the most challenging target for antimicrobial therapies.

The PDI of *E. coli* sensitized by PZnTEP was also evaluated in presence of KI. This inorganic salt has shown impressive bacterial killing when combined with PDI [26,27,39,40,41,42]. The improved therapy results from the formation of a triiodide anion after reaction of iodide anion with traces amounts of O_2_(^1^Δ_g_) (Scheme S1) [26,43]. In this work, we incubated *E. coli* with 100 mM KI; this concentration was selected because no toxicity was found herein (Figure 5B, treatment 5) and previous studies [27]. Also, this inorganic salt was not toxic for *E. coli* exposed to irradiation for 15 and 30 min (Figure S5, treatments 4 and 5). In the presence of the salt, a 7.5 log (99.9999%) reduction in the CFU was found after 15 min irradiation (81 J/cm^2^) (Figure 5B, treatment 6). A similar result was observed after treating the sample for 30 min with white light. Therefore, the combined therapy was at least 2 times more effective than the antimicrobial material alone. In this medium, the interaction of O_2_(^1^Δ_g_) and KI due to light activation of PSs produces biocidal I_2_ or I_3_^-^ improving bacterial inactivation and enlightening an alternative cytotoxic pathway [44].

### 3.7. Photosensitized Inactivation of Bacteria with PZnTEP Coating a Surface

**Figure 6:**
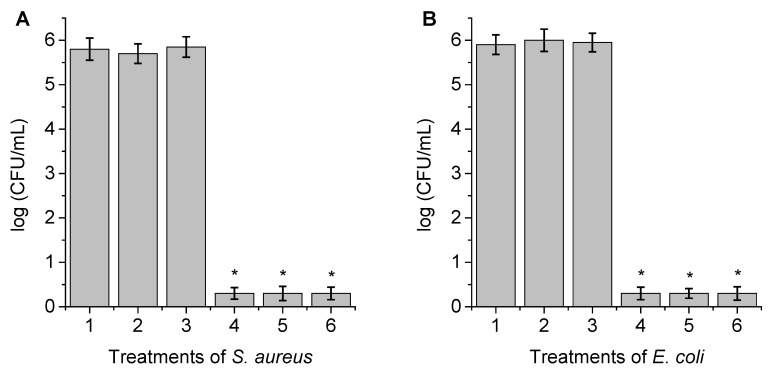
Survival of (**A**) *S. aureus* and (**B**) *E. coli* (~10^6^ CFU/mL) deposited on chamber with PZnTEP attached at the surface and exposed to white light for 30 min: (1) dark control, (2) chamber without PZnTEP and 30 min irradiation, (3) chamber with PZnTEP in the dark, (4) 1st cycle chamber with PZnTEP and 30 min irradiation, (5) 2nd cycle chamber with PZnTEP and 30 min irradiation, (6) 3er cycle chamber with PZnTEP and 30 min irradiation. Inactivation was assessed in a 3D-printed chamber prepared using spin coating. PDI experiments were repeated three times for each bacterial strain in the same chamber to evaluate its reusability (* *p* < 0.05 compared with control).

For these PDI experiments, chambers bearing PZnTEP adsorbed on a PLA surface were prepared by spin coating the PS on top of a coverslip. Prior to deposition, PLA was 3D-printed atop the glass coverslip and melted for a few minutes to achieve homogenous distribution (Figure S1C,D). Subsequently, bacterial suspensions (300 µL in PBS, ∼10^6^ CFU/ mL) of *S. aureus* or *E. coli* were transferred to each container. Control experiments showed that the viability of bacterial cells was not affected by irradiation of chambers without PZnTEP (Figure 6, treatment 2). Also, no toxicity was found in bacteria deposited on the chambers with PS and kept in the dark (Figure 6, treatment 3).

After 30 min irradiation, photoinactivation of bacteria in the presence of the conjugate decreased significantly compared to control (*p* < 0.05). The photodynamic action sensitized by PZnTEP produced over a 5.5 log decrease in bacteria (162 J/cm^2^), which means more than 99.999% bacterial inactivation for both strains (Figure 6, treatments 4–6). Although it is difficult to make a direct correlation, the polymer deposited on the surface of PLA remains as active as 0.5 µM PZnTEP added in cell suspension to inactivate *S. aureus*.

To evaluate the reuse of the surfaces, the photodynamic material was subjected to three cycles of PDI. After each treatment, dead microorganisms were removed and the chamber was rinsed with PBS. A fresh bacterial suspension containing ∼10^6^ CFU/mL was transferred into the chamber and exposed to white light for 30 min. As can be observed in Figure 6 (treatments 4–6) full inactivation was achieved after each cycle with mitigated differences in cell survival. Therefore, the polymer PZnTEP attached to the PLA surfaces can be reused several times, indicating negligible photobleaching and desorption of the PS upon coating the surface. These experiments reveal that the ZnTEP conjugate remains bound to the PLA surfaces with the same PDI potential over the cycles.

In a previous investigation, a porphyrin-fullerene C_60_ dyad substituted by carbazoyl groups was used to obtain electrogenerated polymeric films [45]. The irradiated TCP-C_60_ film produced a 4 log decrease of *S. aureus* survival after 30 min irradiation with white light. Also, a 4 log reduction of *E. coli* viability was obtained using this polymeric film after 60 min of light exposure. Similarly, a polymeric film bearing dendrimeric Zn(II) porphyrin was evaluated as antimicrobial material [23]. When a cell suspension was deposited on this surface, complete elimination of *S. aureus* and a 2 log (99%) reduction in *E. coli* survival was found after 15 and 30 min irradiation, respectively. Moreover, surfaces coated with electroactive phthalocyanines were able to inactivate bacteria mainly when potentiated with KI [42]. Although it is difficult to make a direct comparison, the results indicate that the conjugated polymer PZnTEP shows interesting properties as a photodynamic material to maintain aseptic surfaces.

## 4. Conclusions

The free-base TEP was synthesized by the acid-catalyzed condensation of 4-(ethynyl)benzaldehyde and pyrrole in appropriate yield for a symmetrically *meso*-substituted porphyrin. This porphyrin derivative complexed with Zn(II) acetate to obtain the chelate ZnTEP. The terminal alkyne groups of ZnTEP were subjected to a homogeneous reaction to obtain dynes, which allowed the PZnTEP polymer to form in quantitative yield. Furthermore, xerogel of PZnTEP was obtained by removing the solvent. The polymeric material exhibited microporous structures and spectroscopic characteristics of the ZnTEP were retained in the conjugate. Moreover, photodynamic determinations showed that the porphyrin component in PZnTEP produced O_2_(^1^Δ_g_) and O_2_^•−^. Complete eradication of *S. aureus* cells was found using a low concentration of PZnTEP and a short irradiation time. Furthermore, complete inactivation of *E. coli* was achieved at short irradiation times when PDI was potentiated with KI. Likewise, viable bacteria of *E. coli* were not detected in presence of higher PZnTEP concentration and long irradiation. In addition, PLA surfaces coated with PZnTEP were effective to photoinactivate these bacteria. This surface was reused to eliminate microbial cells with equal efficiency and bacterial cells were efficiently inactivate after each PDI treatment. The results indicate that this conjugated photodynamic polymer represents an interesting antimicrobial photoactive material suitable to eradicate pathogens and can be used to prepare self-sterilizing surfaces. Potential applications of this sensitizing polymer involve the coating of surfaces in hospitals to obtain an aseptic environment, such as surgical rooms, in highly populated public sectors to reduce the microbial load and in self-sterilizing containers activated by natural light to obtain water free of microorganisms.

## Figures and Tables

**Table 1:** Spectroscopic and photodynamic properties of ZnTMP, ZnTEP, and PZnTEP in DMF.

PS	λ^Soret^ (nm)	ε^Soret a^	λ_em_ (nm)	Φ_F_ ^b^	*k*_obs_^DMA^ (s^−1^) ^c^	Φ_Δ_ ^d^
ZnTMP	426	4.07 × 10^5^	608	0.049 ± 0.004	(2.02 ± 0.02) × 10^−2^	0.73 ± 0.03
ZnTEP	428	5.56 × 10^5^	612	0.030 ± 0.003	(1.60 ± 0.02) × 10^−2^	0.57 ± 0.03
PZnTEP	446	-	620	0.008 ± 0.002	(5.30 ± 0.08) × 10^−4^	0.019 ± 0.004

^a^ Molar absorption coefficient (Lmol^−1^cm^−1^), ^b^ fluorescence quantum yield, ^c^ observed rate constants for the photooxidation reaction of DMA, ^d^ quantum yield of O_2_(^1^Δ_g_) production.

## Data Availability

Not applicable.

## Supplementary Materials

The following are available online at https://www.mdpi.com/article/10.3390/antibiotics11010091/s1. Instrumentation; Materials; Figure S1: Photographs of powder dry of PZnTEP, a solution of PZnTEP in DMF, side view and top view of PZnTEP deposited on a chamber for PDI treatments; Figure S2: Model of irradiation systems for steady state photolysis, PDI experiments and emission spectrum of the light source; Figure S3: Absorption spectral changes during the photooxidation of DMA sensitized by ZnTMP, ZnTEP and PZnTEP in DMF at different irradiation times; Figure S4: Absorption spectra changes of NBT photoreduction mediated by NBT + NADH + ZnTEP, NBT + NADH + PZnTEP and NBT + NADH in DMF/water (5%); Figure S5: Survival of bacteria incubated for 30 min at 37 °C in the dark and irradiated with white light for different times; Scheme S1: Reaction of O_2_(^1^Δ_g_) with iodide anions in aqueous media. References [46,47] are cited in the supplementary materials.
