# Supplementary material for: Antimicrobial Photosensitizing Material Based on Conjugated Zn(II) Porphyrins
Source: Antibiotics (Basel). 2022 Jan 12;11(1):91. doi: 10.3390/antibiotics11010091 (PMC8773278; doi:10.3390/antibiotics11010091)
Supplement: Supplementary file 1 [file antibiotics-11-00091-s001.zip › antibiotics-1522258-supplementary.pdf]

Supplementary Materials

Antimicrobial Photosensitizing Material Based on Conjugated Zn(II) Porphyrins

Sofía C. Santamarina[1], Daniel A. Heredia[1], Andrés M. Durantini[1], and Edgardo N. Durantini[1]*

[1]IDAS-CONICET, Departamento de Química, Facultad de Ciencias Exactas, Físico-Químicas y Naturales, Universidad Nacional de Río Cuarto, Ruta Nacional 36 Km 601, X5804BYA Río Cuarto, Córdoba, Argentina.

Table of Contents

1. Instrumentation Page S2

2. Materials Page S2

3. Supporting figures Page S3

4. Supporting scheme Page S5

* Correspondence: edurantini@exa.unrc.edu.ar (E.N.D.)

1. Instrumentation

Proton nuclear magnetic resonance spectra were performed on a FT-NMR Bruker Advance DPX400 at 400 MHz (Bruker BioSpin, Rheinstetten, Germany). Mass spectra were recorded on a Bruker micrOTOF-QII (Bruker Daltonics, Billerica, MA, USA) equipped with an ESI source (ESI-MS). Absorption and fluorescence spectra were carried out on a Shimadzu UV-2401PC spectrometer (Shimadzu Corporation, Tokyo, Japan) and on a Spex FluoroMax spectrofluorometer (Horiba Jobin Yvon Inc, Edison, NJ, USA), respectively. Scanning electron microscopy (SEM) images were obtained with a field emission scanning electron microscope FE-SEM (Σigma Zeiss, Oberkochen, Germany) with a thin Cr film on the sample surface and an acceleration voltage of 5 kV. Containers were printed using a Prusa i3 MK3S 3D printer purchased from Prusa Research (Praga, Czech Republic). A Radiometer Laser Mate-Q (Coherent, Santa Clara, CA, USA) was used to determine the light fluence rates. Steady-state photolysis in solution was performed with a Cole-Parmer illuminator 41720-series (150 W halogen lamp, Cole-Parmer, Vernon Hills, IL, USA). An optical filter (GG455 cutoff filter) was used to select a wavelength range between 455 and 800 nm (44 mW/cm^2, Figure S2 A). Samples were irradiated in a quartz cell of 1 cm path length at room temperature. Cell suspensions were irradiated with a Novamat 130 AF (Braun Photo Technik, Nürnberg, Germany) projector containing a 150 W lamp. A 2.5 cm glass cuvette filled with water without circulation was used to remove the heat from the lamp. The projector was placed vertically with the light beam focused on the 96-well microtiter plate lid, producing a fluence rate of 90 mW/cm^2 (Figure S2B) [46]. A wavelength range between 350 and 800 nm was selected by optical filters [47]. Emission spectrum of the broadband radiation source is shown in Figure S2C.

2. Materials

Chemicals were obtained from Sigma-Aldrich (Milwaukee, WI, USA), which were used without further purification. Organic solvents (GR grade) from Merck (Darmstadt, Germany) were distilled and maintained on molecular sieves. Ultrapure water was obtained from a Labconco (Kansas

City, MO, USA) equipment model 90901-01. Silica gel thin-layer chromatography (TLC) plates (250 microns) were acquired from Analtech (Newark, DE, USA) and silica gel 60 (0.040-0.063 mm, 230-400 mesh) from Merck (Darmstadt, Germany). Tryptic soy (TS) broth and agar from Britania (Buenos Aires, Argentina) were used in microbial cultures. Microtiter plates (96-well) were acquired from Deltalab (Barcelona, Spain). Zn(II) 5,10,15,20-tetra(4-methoxyphenyl)porphyrin (ZnTMP) was synthesized as previously described [24].

3. Supporting figures

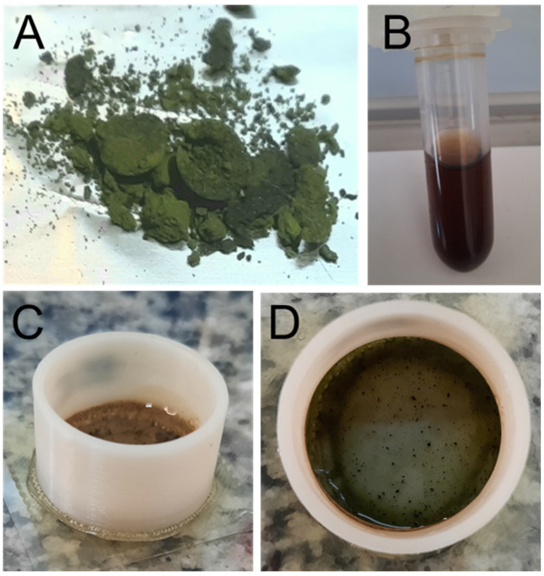

Figure S1. Photographs of (A) powder dry of PZnTEP, (B) a solution of PZnTEP in DMF, (C) side view and (D) top view of PZnTEP deposited on a chamber for PDI treatments.

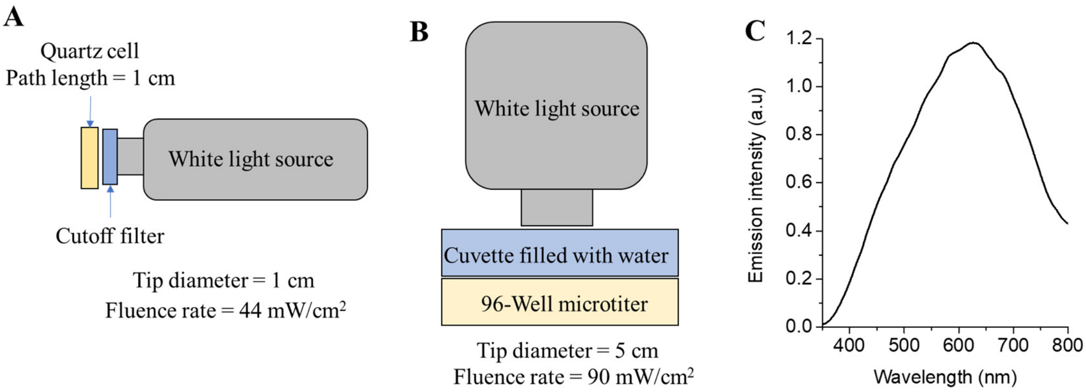

Figure S2. Model of irradiation systems for (A) steady state photolysis, (B) PDI experiments, and (C) emission spectrum of the light source.

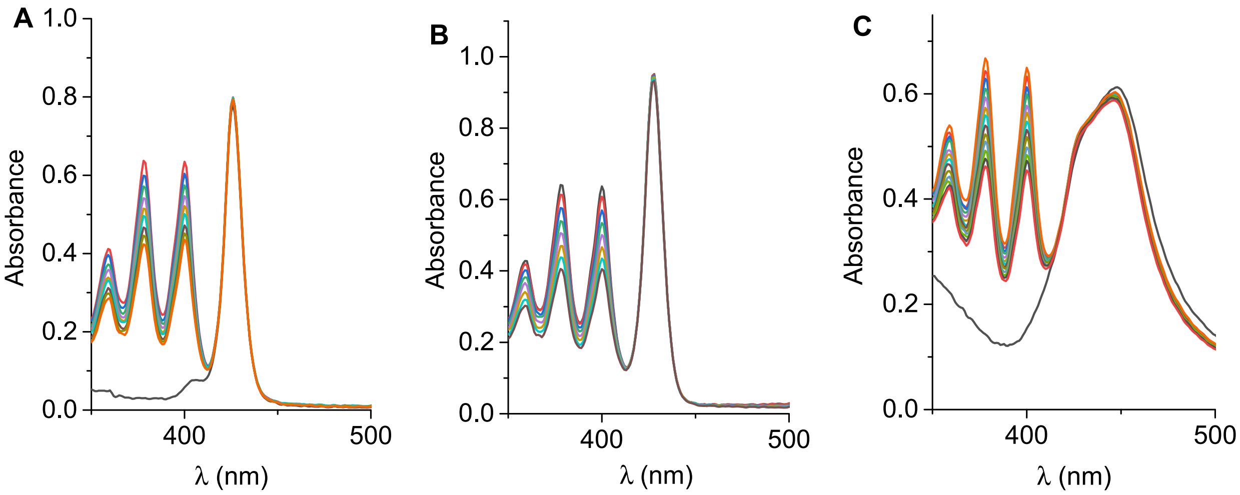

Figure S3. Absorption spectral changes during the photooxidation of DMA sensitized by (A) ZnTMP (Δt = 10 s), (B) ZnTEP (Δt = 10 s) and (C) PZnTEP (Δt = 60 s) in DMF at different irradiation times, λ_{irr} = 455-800 nm.

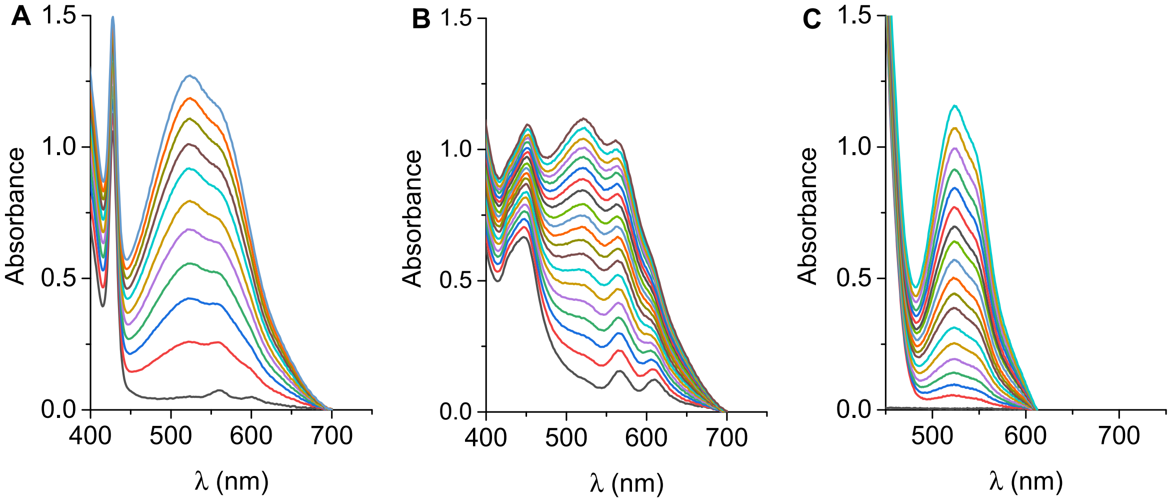

Figure S4. Absorption spectra changes of NBT photoreduction mediated by (A) NBT + NADH + ZnTEP (Δt = 10 s), (B) NBT + NADH + PZnTEP (Δt = 30 s) and (C) NBT + NADH (Δt = 120 s)) in DMF/water (5%), [NBT] = 0.2 mM and [NADH] = 0.5 mM, λ_{irr} = 455-800 nm.

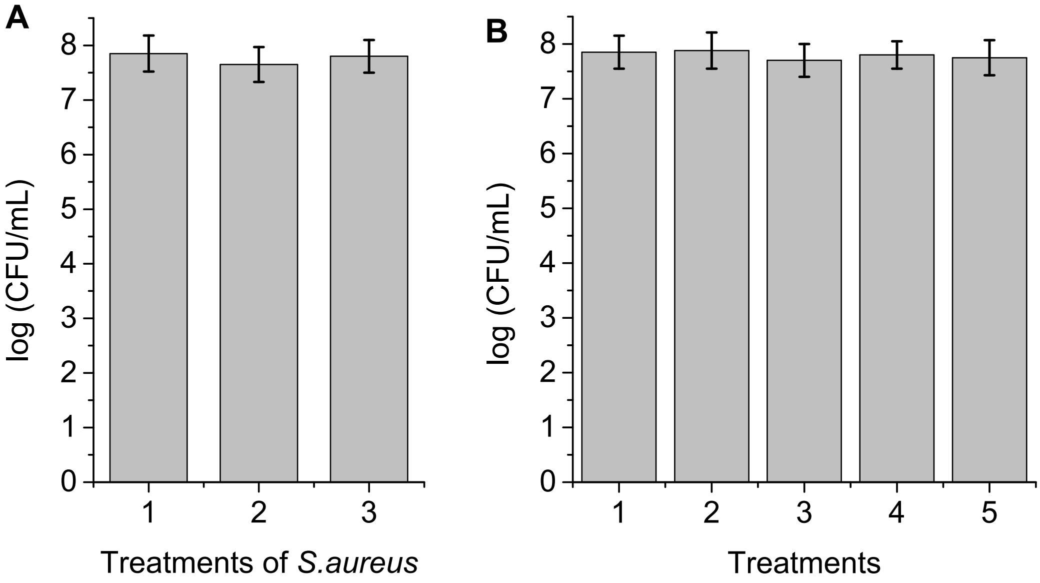

Figure S5. Survival of bacteria (~10^8 CFU/mL) incubated for 30 min at 37 ºC in the dark and irradiated with white light for different times. (A) *S. aureus*: (1) dark control, (2) 15 min irradiation, (3) 30 min irradiation; (B) *E. coli*: (1) dark control, (2) 15 min irradiation, (3) 30 min irradiation, (4) 100 mM KI and 15 min irradiation, (5) 100 mM KI and 30 min irradiation.

$$O_2(^1\Delta_g) + I^- + H_2O \xrightarrow[-OH^-]{} IOOH \xrightarrow{I^-} I_2 + HO_2^-$$

$$I_2 + I^- \longrightarrow I_3^-$$

$$HO_2^- + H_2O \longrightarrow H_2O_2 + OH^-$$

$$H_2O_2 + OH^- + 2I^- + 2H^+ \longrightarrow I_2 + 2H_2O$$

$$I_2 + I^- \longrightarrow I_3^-$$

Scheme S1. Reaction of $O_2(^1\Delta_g)$ with iodide anions in aqueous media [40,43].
